# Understanding the Role of Female Genital Tract Microbiome in Recurrent Implantation Failure

**DOI:** 10.3390/jcm13113173

**Published:** 2024-05-28

**Authors:** Anastasios Lafioniatis, Athina A. Samara, Peter K. Makaritsis, Stefanos Dafopoulos, Sotirios Sotiriou, Konstantinos Dafopoulos

**Affiliations:** 1 Department of Obstetrics and Gynecology, University Hospital of Larissa, 41110 Larissa, Greece; tasoslaf95@gmail.com (A.L.); makaritspeter@hotmail.com (P.K.M.); sn191957@students.euc.ac.cy (S.D.); kdafop@yahoo.com (K.D.); 2 Department of Embryology, Faculty of Medicine, University of Thessaly, 41110 Larissa, Greece; sotiriousoti@yahoo.gr

**Keywords:** microbiome, microbiota, lactobacillus, RIF, IVF

## Abstract

The realization of the role of the microbiome of the female reproductive tract in health and disease has opened numerous possibilities for the scientific examination of the intertwining role between the human host and its microbiota. The imbalance in the composition of the microbial communities of the vagina and uterus is now recognized as a risk factor for many complications in pregnancy and according to the data from numerous studies, it is possible for this imbalance to play a crucial role in creating a hostile endometrial environment, and therefore, contributing to the etiology of recurrent implantation failure. Nevertheless, our current understanding of these complicated biological phenomena is far from complete, and in the future, there needs to be a systematic and thorough investigation of the diagnosis and therapy of this condition. This will enable scientists who engage in the field of assisted reproduction technologies to accurately identify and cure women in whom dysbiosis hinders the achievement of a healthy pregnancy.

## 1. Introduction

Infertility affects about 8 to 12% of reproductive-age couples and has therefore attracted the attention of the global scientific community [1]. Since the birth of the first child through means of In Vitro Fertilization (IVF), assisted reproduction technologies (ART) have become more sophisticated and safe by catching up with the ever-growing scientific knowledge regarding the complex physiological and molecular events behind the miracle of human reproduction. Implantation rates in specialized centers may reach up to 65%. However, in about 35% of the cases, an implantation failure can be encountered, and this can be a great psychological burden for couples and a major economic issue for healthcare systems worldwide. The term recurrent implantation failure (RIF) has been used to mirror the term recurrent pregnancy loss (RPL) and describes the inability of a couple to conceive despite the consecutive embryo transfers of euploid embryos [2]. Although some specialists use the number of two, three, or even four unsuccessful consecutive embryo transfers as a threshold for setting the diagnosis of RIF [3,4], some other scientific groups have expressed their doubt regarding the benefit of using such a one-size-fits-all approach. Instead, they proposed using a more individualized approach by applying prognostic models that take into account the demographic and clinical characteristics of infertile couples [5].

Implantation is an extremely complex phenomenon that can be influenced by a lot of factors [6]. It is the result of the successful cross-talk between the blastocyst and the endometrium and is therefore dependent on the good quality of the embryo, regarding its genetic composition, and the receptivity of the endometrium, which refers to the ability of the endometrial lining to accept and nurture the blastocyst and result, therefore, in a successful pregnancy [7]. The status of the endometrium on the cellular level is tightly regulated by a complex set of molecular events involving endocrine, paracrine, anatomic, and immunologic factors [8,9]. The immunological imbalance has attracted a lot of attention in recent years. In many women with difficulties to conceive or maintain a viable pregnancy, an imbalance of the constituents of the native and the adaptive immune system is often observed [10]. Such a state of immunologic imbalance is a condition called chronic endometritis (CE) which was often underdiagnosed in the past because of the scarcity of clinical symptoms and the fact that diagnosing CE requires the acquisition of endometrial tissue and histopathologic and immunohistochemical examination with anti-CD 138 monoclonal antibody for the detection of plasma cells [11,12,13,14,15,16,17].

Although in the past the relationship between human and the microorganisms that reside in various loci of the human body was overlooked or superficially examined, there is now an ever-growing amount of evidence that the interaction between the human host and its microbiome is far more complex and important than people dared to imagine [18,19,20]. An important factor that contributed to this radical change is the introduction of more sensitive molecular methods such as the next generation sequencing (NGS) techniques. Numerous reports link the human microbiome, which some call “the other human genome”, with a variety of pathological states. Having this in mind, the role of the microbiome is thoroughly investigated in order to determine its role in gynecological and obstetric disease [21]. The imbalance in the relative microbial composition in the female genital tract is now considered as a significant contributing factor in diseases such as infertility, preterm labor, RPL, and bacterial vaginosis [22,23,24,25,26,27]. The healthy microbiome of the genital tract, a state called eubiosis, plays a protecting role by competing with other pathogenic species and creates a favorable chemical environment for the function of the female reproductive system.

It is subsequently hypothesized that the deviation in the composition of the microbiome, i.e., dysbiosis [28] may play a key role in the development of CE and the disturbance in the immunological and hormonal response of the endometrial cells [29,30,31,32,33,34]. The result of this pathological procedure is the altered receptivity of the endometrium and as a consequence, the implantation failure. The aim of the present review article is to elucidate the potential role of female genital tract microbiome in recurrent implantation failure, based on the current literature data.

## 2. The Microbiome of the Female Reproductive Tract

It has been known for a long time that the bacteria of the genus *Lactobacillus* are a significant constituent of the vaginal microbiome. Lactobacilli are the most abundant bacteria in the vagina and keep the vaginal pH < 4.5 by metabolizing the glycogen of the epithelial cells into lactic acid [35]; they generate hydrogen peroxide [36] and bacteriocins creating an unfavorable environment for various pathogenic species of bacteria, protozoa, and fungi, and thus they prevent the colonization of the vagina [37]. After menstruation or the post-coital exposure of the vaginal epithelium to seminal fluid, the protective effects of Lactobacilli are reduced creating the conditions that are required for a change in the vaginal microbiome. By using molecular techniques, scientists were able to characterize the microbial species inhabiting the vagina. Five groups were identified, called community state types (CSTs), four of which, namely CSTs I, II, III, and V were *Lactobacillus*-dominant (>90%), and one (CST IV) was characterized as Lactobacillus non-. In *Lactobacillus*-dominant microbiomes, *Lactobacillus crispatus*, *Lactobacillus gasseri*, *Lactobacillus inners*, and *Lactobacillus jensenii* were predominantly harbored in CSTs I, II, III, and V accordingly. In CST IV, on the other hand, a dominance of non-Lactobacilli genera is observed such as *Prevotella*, *Gardnerella*, *Streptococcus*, *Bifidobacterium*, *Enterococcus*, and *Staphylococcus* [38]. 

*Lactobacillus*-dominant CSTs are relatively stable, although some transition may occur. In contrast, CST IV is highly variable often changing in one of the other CSTs. It is important, therefore, to note that the characterization of the vaginal microbiome can give us only a temporary profile. The reduction in the number of lactobacilli creates a niche for other species to multiply and colonize the vagina, which would otherwise be kept under control by the lactobacilli. This change can be caused by lifestyle factors and the use of antibiotics. This altered microbial composition has emerged through studies as a major factor for the creation of bacterial vaginosis and recurrent Candida infections. Bacterial vaginosis is also recognized as a risk factor for many obstetric complications such as preterm labor, the premature rupture of membranes, and other negative obstetric outcomes [33,39]. It should be noted that not all Lactobacilli species may play a protective role as CST III characterized by *Lactobacillus iners* is associated with an increased risk of developing bacterial vaginosis [2]. This could be explained by the difference in the metabolic profiles, as it is known that *Lactobacillus iners* produces only the L isomer of lactic acid and not the D isomer, whose significance for vagina health has been for long a topic of much discussion, and does not produce hydrogen peroxide [40,41]. Ethnical and geographic characteristics may also contribute to the microbial composition as Lactobacilli-dominant CSTs were found more frequently in Caucasian or Asian Women whereas Lactobacilli non-dominant CST IV was more common among Hispanic and African American women [42]. 

For a long time, it has been hypothesized that the upper part of the female genital tract, namely the uterus and the fallopian tubes, were sterile. The application of newer techniques that did not rely on culture media revealed the existence of a microbiome colonizing the inner lining of the uterine cavity [20,43,44]. The abundance of microbial cells in the uterine cavity was significantly lower compared with the vagina, as it was found that it hosted four magnitudes of order fewer microorganisms. In these studies, *Lactobacillus* also played a dominant role supporting the idea that the microbiome of the genital tract displays a continuum between its upper and lower portions [45]. On the other hand, there are, however, reports that disagree on the actual composition of the uterine microbiome arguing that the results of the former studies are a result of sample contamination [46] while others even doubt the mere existence of such a microbiome. In another study where samples were taken from the endometrial cavity after a hysterectomy for leiomyomas, there were no microorganisms detected [47]. 

Moreover, some researchers claim that the microbial composition of the uterine cavity fluctuates reflecting the cyclic hormonal changes that take place during the menstrual cycle [48]. This is, however, a hypothesis that is contradicted by reports where no such changes were observed [49].

Further along the upper female genital tract and specifically in the fallopian tubes, there is a greater microbial diversity with Lactobacilli no longer being the dominant species while *Lactobacilli genera*, *Acinetobacter*, *Vagococcus*, and *Sphingovium* comprise a significant fraction of the microbiota. Lastly, the peritoneal fluid aspirated from the pouch of Douglas was characterized by a lack of Lactobacilli and a great diversity but not exactly the same composition as that of the fallopian tubes [45,50]. 

## 3. Diagnostic Procedures—Sampling Techniques

There is a wide variety of techniques for obtaining a suitable sample for the analysis of the female reproductive tract microbiome. The most common method is the collection of vaginal fluid [32,51]. According to some researchers, the microbial community of the vagina reflects the one residing in the endometrial cavity. Taking a good sample is easy and runs a low risk of contamination with the microbial species of the vulvar region. Another method that has been described by some authors is the collection of mucus from the cervical opening during a colposcopic examination [52]. The rationale behind this procedure is the thought that the cervical mucus creates an important obstacle for the microbiota of the vagina preventing many of them from entering the uterine cavity. As a result, the microbial composition of this particular area reflects better the intrauterine microbiome. 

On the other hand, some researchers support that there can be no substitutes for the immediate sampling of the uterine cavity for the screening of its microbial environment. Some researchers have described a technique in which the endometrial cavity is flushed with sterile saline after the insertion of a plastic catheter which is connected on its one end with a syringe [53] while others aspirate the already existing fluid without flushing the uterine cavity [51]. Another team has described a method, which is quite similar to that of a regular pipelle, which aims at the retrieval of small fragments of endometrial tissue which will then undergo molecular analysis [54,55,56,57]. A major drawback of these two techniques is the fact that the endometrial microbiome is theorized to be made up of far fewer microbial cells than the neighboring vaginal microbiome, making these sampling techniques extremely liable to contamination. In order to carry out these procedures, one should be very careful during sampling by using sterile protective equipment such as glove and gown, carefully sterilizing the cervicovaginal area so as not to introduce foreign microbiota from the vagina into the uterus, and always have in mind not to touch the vaginal walls during the withdrawal of the sampling catheter. Nevertheless, our opinion is that in order to acquire a reliable sample so as to be able to cure a possible disturbance in the uterine microbiota and achieve a clinical pregnancy, uterine fluid aspiration seems to be at the moment the best option.

The emergence of highly sensitive molecular techniques has enabled us to better understand and characterize the diversity of the human microbiome. Since the time that the Amsel criteria and the Nugent score were used, the historical significance of which no one can deny, the rapid progress of biotechnology has provided us with molecular methods that are able to overcome the diagnostic limitations posed by conventional microscopy and microbial cultures and have been applied for many decades in everyday clinical practice. Real-time PCR, also known as qPCR (avoiding to be confused with reverse transcriptase PCR—RT-PCR), has been utilized in order to detect certain bacterial genera and species utilizing the amplification of certain sequences that are specific for certain microorganisms [58]. There have also been reports of using micro-array identification methods which employ the property of the complementary binding of species-specific sequences onto selected probes [59]. NGS techniques are also a breakthrough that has driven metagenomic analysis forward [60]. Although the term NGS contains a wide variety of techniques, the general principle behind these methodologies is a rapid sequencing of DNA fragments that are present in a specimen and the subsequent alignment of the resulting sequences and the construction of a profile which then through complex bioinformatic analysis is matched with a specific microbial mixture. Furthermore, a meta-transcriptomic approach is mentioned to be applied in one report [20].

Molecular-based techniques are generally more sensitive than microscopy and culture-based methods; however, one should always consider that some of these may be quite cumbersome while others require a high level of expertise both for handling the equipment and managing and interpreting the data that come out of these analyses. Another important factor is the cost of these methods, regarding both the reagents and the analytical devices, rendering the use of these techniques quite difficult. It is therefore imperative that more experience in applying these methodologies needs to be acquired and the costs be lowered so that widespread application can take place. 

The most common molecular target that has been used for the identification of the microorganisms that consist of the female genital tract microbiome is the 16S rRNA [52,60,61,62,63]. This particular molecule is the nucleic acid component of the small subunit of the 70S ribosome of prokaryotic organisms. Although the sequence of the 16S rRNA is highly conserved among microbial species, there are certain hypervariable regions. These regions are nine, named V1–V9, ranging between 30 and 100 bp, and are involved in the secondary structure of the small subunit. The degree of conservation varies to a great extent between hypervariable regions with more-conserved regions correlating to higher taxonomy whereas less-conserved regions correlate to lower levels such as genus and species [59]. Those regions within the ribosomal RNA serve as molecular signatures and are employed by modern molecular techniques in order to investigate the metagenomics of the various compartments of the human body, and in our case, the microbial communities that reside in the various niches of the female reproductive system. One significant advantage of the procedures that target these sequences in comparison to conventional diagnostic techniques such as cultures and microscopy is the fact that even in low concentrations, a wide variety of microorganisms can be identified, and this is particularly helpful for species that require specific conditions for their culture, making their isolation and characterization extremely difficult.

As far as the statistical analysis is concerned, there is heterogeneity in statistical analysis methods among the published articles which can be explained by the diversity of the analytical procedures, and therefore, the correspondent statistical packages required for data analysis. The most common measures that the authors used in the statistical analysis of their samples were the α and β diversity. Alpha diversity measures can be seen as a summary statistic for a population (within sample diversity) whereas beta diversity measures are the measures of similarity or dissimilarity between populations (between samples).

## 4. Microbiota and RIF

The dysbiotic state of the vaginal microbiome can be a result of many factors including infections, vaginal douches [64], certain diets [65] as well as the prolonged use of antibiotics and other types of medication [66]. A higher diversity in the microbial population of the vagina was detected in women with RIF when compared to control women [32]. An abundance of genera and species related to bacterial vaginosis such as *Gardnrella*, *Atopobium*, *Prevotella*, *Megasphaera*, *Burkholderia*, and *Sneathia*, and reduced numbers of Lactobacilli were reported [67] in the women affected by RIF in contrast to controls by several independent studies [55,68,69,70,71,72,73]. As far as the *Lactobacillus* species are concerned, a relative predominance of *Lactobacillus helveticus* and not of *L. iners*, *L. crispatus*, *L.jensii*, and *L. gasseri* was shown to exist in the women with RIF [68]. At the same time, the presence of L.iners in the microbiome of the women with RIF suggests a more complex relationship [74] and the need for a clarification of the exact role of individual Lactobacilli species in the future [2,75,76].

As mentioned above, the uterine cavity harbors far fewer microorganisms and specifically, about 10,000 times fewer microorganisms in comparison to the vagina [45]. This significantly smaller biomass and the relative inaccessibility of the uterine cavity without risking the contamination of the specimen is the reason why no consensus has been reached regarding the composition of the healthy uterine microbiome [77,78]. In a study of 141 women with RIF, 121 individuals had *Lactobacillus* non-dominant microbiomes, and high numbers of *Streptococcus*, *Staphylococcus*, *Neisseria*, and *Klebsiella* were reported [2] which are known to disrupt the normal endometrium [11]. Another bacterial genus linked to RIF when present in the endometrium is that of Bifidobacterium [79]. Another set of studies has shown a drastic decrease in Lactobacilli and an abundance of *Dialister*, *Prevotella*, *Gardnerella*, and *Anaeroccus* in women with chronic endometritis [28,67]. The disruption of the healthy uterine microbiome is thought to result in chronic endometritis [77] and triggers a pro-inflammatory response that renders the endometrium unfavorable for implantation [22,32,80]. What we need to mention, however, is the fact that there are studies that failed to report a difference between the women with dysbiotic and eubiotic communities as far as the IVF success rates are concerned [55,81]. Finally, there are even reports in which there is no difference in the microbial composition between the women who underwent IVF and got pregnant and those who did not. Some studies even showed that the microbiomes in the women who failed to become pregnant after ART therapies were *Lactobacillus*-dominant, a characteristic that many recognize as a beneficial factor.

## 5. Genital Tract Dysbiosis Management

When CE is established as a contributing factor for the RIF that affects a couple and taking into consideration the role of female tract organisms in the pathogenesis of CE, antibiotics are considered as the first line of treatment. One study enlisted patients with chronic endometritis who were given oral antibiotics particularly a 14-day course of 100 mg doxycycline twice a day, and the result of the treatment was monitored using hysteroscopy and biopsy. The patients with persisting positive biopsies underwent treatment with a further antibiotic regimen consisting of ciprofloxacine 500 mg and metronidazole 500 mg both taken twice a day [82]. In the women who were cured, a significant increase in pregnancy and live birth rates was observed in comparison to the women not responding to the antibiotic treatment. In another study that enrolled 421 women affected by RIF, a high prevalence of CE was observed. After applying a similar antibiotic regimen to the one mentioned above, better IVF and pregnancy outcomes were observed. Analogous results have come from several studies [3]. Some researchers have chosen a more targeted approach regarding the antibiotics used. When Gram-positive microbes were detected, a combination of Amoxycilline and Clavulanate was used for 8 days [3]. Gram-negatives were treated with ciprofloxacine for 10 days, and atypical bacteria were treated with 1 g Josamycin twice a day for 12 days with the addition of Minocyclin in persistent cases [3]. Nevertheless, we have to point out the presence of the reports that did not show a significant improvement in implantation rates as well as clinical pregnancy rates as a result of CE treatment [7]. 

In an interesting study, oral administration was supplemented by intrauterine antibiotic infusion [83]. However, no differences in clinical pregnancy rates were documented. This was attributed to a possible disturbance in the endometrial environment caused by the intrauterine infusions. To the best of our knowledge, there are no studies investigating the effect of local antibiotic treatments. This is an issue that needs to be addressed in the future, as the local application of antimicrobials may prevent any systemic adverse effects of these medications and the disruption of microbial composition in other areas of the body such as the skin or the gastrointestinal system.

One other aspect that we also need to take into consideration, is that antimicrobial treatment may be just one of the interventions that we can apply to reestablish a more balanced microbiome. Pre- and probiotics are such a modality that has recently attracted a lot of scientific attention [84,85]. In a study about the vaginal supplementation of intravaginal probiotics and namely lactobacilli, no benefit was observed regarding implantation rates or clinical pregnancies. What was, however, observed was a decrease in the miscarriage rates of the treated patients [86]. On the other hand, there is a report that showed a positive impact on IVF success rates in women whose disturbed endometrial microbiome was restored using probiotic and antibiotic therapy prior to embryo transfer. Among the newer therapeutic modalities, we will also have to refer to autologous platelet-rich plasma (PRP), a method that creates new perspectives in treating CE and RIF [87].

## 6. Future Perspectives

When studying the existing literature regarding the role of *Lactobacillus* and RIF, several issues are encountered that will have to be answered in the future. As mentioned above, there is still a debate concerning not only the consistency of the uterine microbiome but also its mere existence. Therefore, carefully designed large-scale studies need to be executed in order to determine the optimal specimen whether that is the vaginal fluid, the endometrial fluid, or even endometrial tissue, and the best sampling method that will have to be both sensitive and not prone to contamination. This way, a common practice can be established in order to definitely answer the question of uterine microbiome, eliminating any conflicting results that may arise due to different methodologies. Another issue that needs to be addressed is the fact that many of the mechanisms that control the interaction between the human host and its microbiome are still largely unknown [40]. 

Table 1 summarizes the main outcomes of the most important studies regarding the role of the microbiome of the female genital tract in reproduction. We need to perform studies in order to elucidate the complex molecular factors behind the effect that microorganisms exert on the human organism and vice versa. By obtaining a better understanding of the molecular biology of this reciprocal relationship, we will be able to develop more targeted therapies and interventions. Moreover, the time of sampling and the most suitable method of the molecular analysis of the obtained specimens need to be clarified. We also have to consider the aspect of cost-effectiveness. The development of guidelines can also be of great assistance to the professionals engaged in ART [70]. While the identification of infertile couples in whom the dysbiotic endometrium contributes to RIF is important, equally significant is the implementation of a safe and effective treatment in order to overcome this adversity and achieve the desired outcome which is the birth of a healthy child.

A consensus needs to be made on the antimicrobial agents, the dose, and the number of days required to reestablish a healthy microbiome. This can be achieved by randomized clinical trials which recruit a large number of patients. The use of antimicrobial agents also needs to be specific and applied only when necessary, taking into consideration the high rates of microbial resistance that have emerged due to the reckless use of antibiotics. Additionally, another aspect that needs to be investigated is whether there is a place for pre- and probiotics in establishing and maintaining a healthy microbial population in the female genital tract, thus creating a more suitable and favorable environment for the implantation of the embryos and the preservation of an uncomplicated pregnancy [79].

## 7. Conclusions

The realization of the role of the microbiome of the female reproductive tract in healthy and diseased states has opened numerous possibilities for the scientific examination of the intertwining role between the human host and its microbiota. The imbalance in the composition of the microbial communities of the vagina and uterus is now recognized as a risk factor for many complications in pregnancy, and according to the data from numerous studies, it can possibly play a crucial role in creating a hostile endometrial environment, and therefore, contributing to the etiology of RIF. Nevertheless, our current understanding of these complicated biological phenomena is far from complete, and in the future, there needs to be a systematic and thorough investigation of the diagnosis and therapy of this condition. This will enable scientists who engage in the field of ART to accurately identify and cure women in whom dysbiosis hinders the achievement of a healthy pregnancy.

## Figures and Tables

**Table 1:** Main studies’ outcome summary.

First Author (Year)	Country	Study Group	Sampling Method	Outcomes
Ravel et al. [38] (2011)	U.S.A.	A total of 396 women (98 White, 104 African American, 97 Asian, and 97 Hispanic)	Vaginal swabs	In total, 5 microbial communities (I–V) were characterized. Communities I, II, II, and V are *Lactobacillus* dominated while IV is not. In African American and Hispanic women, there is a greater prevalence of group IV.
Moreno et al. [49] (2016)	Spain	A total of 35 fertile and 35 infertile women	Endometrial and vaginal aspirates	NLD microbiomes correlated with negative reproductive outcomes.
Winters et al. [47] (2019)	Italy	A total of 25 women who underwent hysterectomy (23/25 for leiomyomas and 2/25 for endometrial hyperplasia)	Cervical, vaginal, rectal, and endometrial samples following hysterectomy	Bacterial load was detected in 15/25 (60%) of the patients and was dominated by *Acinetobacter*, *Pseudomonas*, and *Cloacibacterium* and not *Lactobacilli.*
Kitaya et al. [51] (2019)	Japan	A total of 28 women with RIF and 18 women undergoing the first IVF attempt	Endometrial fluid and vaginal secretion	No differences in the composition of vaginal secretions between the two groups. There was a significant difference in the microbial composition of endometrial fluid between the RIF group and controls. Burkholderia was detected only in the RIF group.
Fu et al. [32] (2020)	China	A total of 27 women with RIF and 40 controls	Vaginal swabs	Vaginal microbiota shows greater diversity in the patients with RIF than controls and lower abundance in *Lactobacillus* which is correlated with pregnancy rates.
Kong et al. (2020)	China	A total of 475 women undergoing embryo transfer	Vaginal swabs	Age, endometrial thickness, the reduction in *Lactobacillus*, and the overgrowth of *Gardnerella*, *Atopobium*, and *Prevotella* possessed strong connection with the success of IVF.
Diaz-Martinez et al. [68] (2021)	Spain	A total of 48 women with RIF	Endometrial and vaginal swabs	No differences in microbial diversity between the women who got pregnant and those who did not. There were differences in the taxonomic classification.
Ichiyama et al. [55] (2021)	Japan	A total of 145 women with RIF and 21 controls	Vaginal and endometrial samples	Microbial diversity was higher in the RIF group and *Lactobacillus* abundance was lower.
Sola-Leyva et al. [20] (2021)	Spain	A total of seven healthy women	Endometrial biopsy	*Lactobacillus* levels fluctuate during the menstrual cycle and in healthy women the endometrium was not lactobacillus-dominated.
Moreno et al. [73] (2022)	Spain	A total of 342 infertile women	Endometrial fluid and biopsy	High *Lactobacillus* content is associated with live pregnancy rates. Dysbiotic microbiomes are associated with unsuccessful outcomes.
